# Emerging strategies for spinal cord injury repair: stem cells, extracellular vesicles, biomaterials, and neuromodulation

**DOI:** 10.3389/fbioe.2026.1870955

**Published:** 2026-07-15

**Authors:** Bing Wu, Xiangchang Ying, Jiayi Dou, Han Zhang, Chen Chen, Junwei Feng, Jiale Zhang, Binbin Tang, Lianguo Wu, Liqiang Dong, Tingyuan Lai, Zhongcheng An, Hao Wei

**Affiliations:** 1 The Second School of Clinical Medicine, Zhejiang Chinese Medical University, Hangzhou, Zhejiang, China; 2 Department of Orthopedics, The Second Affiliated Hospital of Zhejiang Chinese Medical University, Hangzhou, Zhejiang, China; 3 Department of Spine Surgery, Xinhua Hospital, Shanghai Jiao Tong University School of Medicine, Shanghai, China

**Keywords:** biomaterials, clinical translation, extracellular vesicles, neural repair, neuromodulation, spinal cord injury, stem cell therapy

## Abstract

Spinal cord injury (SCI) is a devastating condition that frequently results in permanent disability. Repair and functional recovery after SCI are hindered by the complexity of its pathophysiology and the inherent challenges of neural regeneration within the central nervous system. Despite decades of research aimed at elucidating its pathological mechanisms and developing effective strategies to promote axonal regeneration and circuit rewiring, successful therapeutic outcomes remain limited. Recent advances in bioactive materials and stem cell technology have shifted the research focus from solely stimulating corticospinal tract regeneration toward building intermediate neural networks to facilitate neural repair and circuit reconstruction. Concurrently, emerging technologies that modulate neural activity through physical modalities, such as electrical, magnetic, and ultrasonic stimulation, are rapidly evolving. Among these emerging strategies, several—including stem cell therapy, biomaterial-based interventions, and electromagnetic stimulation—have advanced to clinical trials, with some already entering clinical practice. This review summarizes the major contemporary strategies and research advances in SCI repair. It details the mechanisms and latest developments in stem cell and extracellular vesicle therapy, biomaterial applications, and neuromodulation techniques. Finally, it discusses future therapeutic directions and ongoing challenges in clinical translation.

## Graphical Abstract

**Figure:**
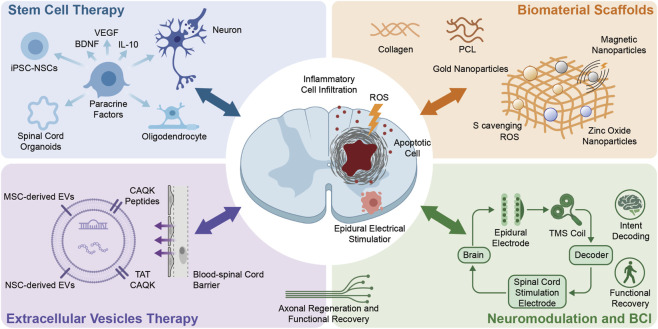
Stem cells, extracellular vesicles, biomaterials, and neuromodulation act synergistically to remodel the inhibitory microenvironment, promote neural regeneration and circuit reconstruction, and improve functional recovery after spinal cord injury.

## 1 Introduction

Spinal cord injury (SCI) is a devastating neurological condition associated with high rates of disability. It causes persistent neurological deficits, markedly reduces patients’ quality of life, and imposes a substantial burden on families and society (Holmes, 2017). According to estimates from the World Health Organization, approximately one million people sustain SCI worldwide each year from various causes, and its incidence continues to increase (Trinka et al., 2023).

It is also important to note that non-traumatic spinal cord injuries caused by severe spinal degeneration, such as intervertebral disc degeneration and osteoporotic compression fractures, are becoming increasingly common. These pre-existing degenerative pathologies create a chronically inflamed, hypoxic, and biomechanically altered local microenvironment. Unlike acute traumatic injury, this chronically hostile niche makes subsequent neural regeneration considerably more difficult, underscoring the urgent need for therapies that can effectively remodel the injured microenvironment.

**FIGURE 1:**
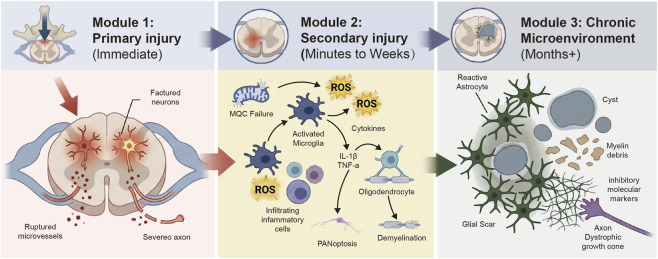
Pathological progression of spinal cord injury (SCI) from primary injury to secondary injury and chronic inhibitory microenvironment formation. Primary injury involves immediate mechanical disruption of spinal cord tissue and blood vessels. Secondary injury is characterized by reactive oxygen species (ROS) accumulation, neuroinflammation, mitochondrial quality control (MQC) failure, demyelination, ferroptosis, and PANoptosis. Chronic SCI is marked by glial scar formation, cystic cavitation, myelin debris accumulation, and axonal dystrophy, which collectively restrict neural regeneration and functional recovery. Abbreviations: SCI, spinal cord injury; ROS, reactive oxygen species; MQC, mitochondrial quality control; PANoptosis, pyroptosis-, apoptosis-, and necroptosis-associated inflammatory cell death.

The pathophysiology of SCI primarily involves two phases: primary and secondary injury, as shown in Figure 1. Primary injury mainly results from persistent compression or tearing of blood vessels and spinal cord tissues following external forces applied to bone, intervertebral discs, or ligaments (Freund et al., 2019; Eli et al., 2021). Traditionally, secondary injury pathology has focused on localized oxidative stress, inflammation, and neuronal apoptosis at the injury epicenter (Anderson et al., 2022; Bertels et al., 2022; Khachatryan et al., 2022). Recently, this paradigm has evolved to encompass a highly interconnected network of organelle dysfunction and non-apoptotic regulated cell death (RCD) modes, particularly mitochondrial quality control (MQC) failure, ferroptosis, PANoptosis, and cuproptosis (Wang Q. et al., 2025; Zhang et al., 2026). Rather than acting as isolated events, these newly identified death pathways are tightly coupled with the metabolic state of the injured niche, collectively driving neuroinflammatory amplification, lipid peroxidation, and progressive white matter degeneration (Lou et al., 2024; Li et al., 2026). Redefining SCI pathophysiology through the lens of these interactive metabolomic and death networks not only reshapes our understanding of microenvironmental remodeling, but also uncovers novel biophysical targets for synergistic engineering interventions.

Notably, “lesion-remote astrocytes” (LRAs) have emerged as crucial orchestrators of white matter repair. Following injury, LRAs secrete the matricellular protein CCN1, which binds to microglial SDC4 receptors to reprogram their lipid metabolism, enabling the efficient digestion of lipid-rich myelin debris (McCallum et al., 2026). Depletion of astrocyte-derived CCN1 impairs this debris clearance, leading to abnormal lipid accumulation, heightened neuroinflammation, and hindered recovery. These findings suggest that future synergistic therapies may need to extend beyond the physical injury cavity to engage remote glial networks and improve microenvironmental remodeling (Takata et al., 2026).

While standard clinical interventions—including acute surgical decompression, pharmacological neuroprotection, and subsequent supportive rehabilitation—are indispensable for stabilizing the injury and preventing early secondary propagation, they remain general strategies that fail to trigger active parenchymal regeneration. This clinical bottleneck is primarily rooted in the complex, chronically hostile microenvironment that persists after the acute phase. Consequently, to translate passive neuroprotection into active neurological restoration, the surgical management paradigm must evolve. The physical cavities opened during mandatory decompressive surgery should not merely be closed, but leveraged as ideal spatial windows for the *in situ* administration of advanced biomaterials. In recent years, the interdisciplinary convergence of fields such as stem cell technology, biomaterial engineering, and neuromodulation has driven a profound shift toward multimodal paradigms. This integration allows for the localized retention of regenerative agents (such as stem cells and extracellular vesicles) within the material-filled surgical cavity, while post-operative therapeutic pathways can be further augmented by neuromodulation to rewire newly formed circuits.

This review aims to summarize recent major therapeutic strategies and research progress in SCI repair. It focuses on emerging approaches including stem cell and extracellular vesicle therapy, biomaterial applications, and neuromodulation techniques. These core elements may function synergistically: stem cells provide robust paracrine signaling, biomaterial scaffolds offer structural support and spatiotemporal drug delivery, extracellular vesicles enable targeted molecular delivery across biological barriers, and neuromodulation facilitates active circuit stimulation and functional restoration. Together, these strategies may help modulate the inhibitory injury microenvironment to promote neural regeneration and functional recovery. Furthermore, it discusses future directions and persisting challenges, with the goal of informing ongoing theoretical innovation and clinical practice in the field.

## 2 Current clinical management and translational bottlenecks

### 2.1 Surgical decompression and stabilization: reconstructing the physical niche

Acute surgical decompression and instrumented spinal stabilization remain the undisputed clinical gold standards to alleviate mechanical pressure, restore spinal canal patency, and prevent ongoing primary tissue tearing (Fehlings et al., 2012). Historically, the optimal timing for surgical intervention remained highly controversial due to conflicting data from early single-center prospective cohort trials and retrospective series (Vaccaro et al., 1997; La Rosa et al., 2004). This clinical uncertainty has been substantially clarified by the landmark Surgical Timing in Acute Spinal Cord Injury Study (STASCIS). (Fehlings et al., 2012). This multicenter investigation provided influential evidence supporting early surgical decompression within 24 h after injury, showing an increased likelihood of neurological improvement on the ASIA Impairment Scale (AIS) at 6 months without a clear increase in perioperative risks or acute inpatient complication rates. Nevertheless, the optimal timing and expected benefit of decompression should still be interpreted in the context of injury severity, patient heterogeneity, surgical feasibility, and institutional experience.

However, while acute mechanical stabilization successfully restores macro-anatomical alignment, it inevitably leaves a localized physical cavity filled with inhibitory myelin debris and a chronically hostile, ischemic microenvironment. This macro-level surgical access, therefore, establishes a critical temporal and spatial window. Instead of treating surgery as mere structural closure, this intervention should be leveraged as a unique micro-anatomical route for the *in situ* administration of emerging biomaterial scaffolds or cellular hubs directly into the lesion site, thereby maximizing local retention and bypassing systemic pharmacokinetic barriers.

### 2.2 Current pharmacotherapy and molecular barriers: the delivery bottleneck

Systemic pharmacotherapy for acute SCI has historically focused tightly on mitigating secondary injury cascades, such as neuroinflammation, microvascular failure, and localized oxidative stress (Fehlings et al., 2012). This approach has historically been represented by high-dose methylprednisolone sodium succinate (MPSS) pulse therapy, which was widely investigated and incorporated into some clinical protocols following the Second National Acute Spinal Cord Injury Study (NASCIS-2) (Bracken et al., 1990). Nevertheless, the clinical translation of systemic pharmacotherapy remains highly controversial, leading clinical bodies to classify steroid administration as a discretionary option rather than a rigid standard of care. This hesitation stems from the suboptimal bioavailability of systemic drugs at the injury epicenter and the associated risks of severe, systemic adverse events, including pulmonary and infectious complications (Fehlings et al., 2012).

The fundamental barrier resides in the mode of administration; systemic intravenous injection is heavily restricted by the blood-spinal cord barrier (BSCB) and risks rapid systemic clearance. This clinical limitation highlights a crucial delivery bottleneck, directly necessitating advanced, target-engineered vehicles—such as extracellular vesicles (EVs) or hydrogel-tethered delivery vectors—capable of actively crossing biological barriers for localized, spatiotemporally controlled molecular regulation.

### 2.3 Conventional rehabilitation vs. active neuromodulation: the circuit rewiring challenge

Chronic-stage therapeutic management heavily relies on standard physical therapy and standardized post-operative rehabilitation regimens to promote long-term functional adaptation (Fehlings et al., 2012). Clinical trial frameworks established by historical investigation series, including the Sygen multicenter acute SCI study, emphasize that the vast majority of natural neurological recovery is bound to these structured subacute and chronic timelines (Geisler et al., 2001). Furthermore, standard management guidelines dictate that these physical therapies must be supported by rigorous, hyperacute medical stabilization, such as induced hypertensive therapy (maintaining mean arterial pressure >85 mmHg), to preserve perfusion to vulnerable neural circuitry (Hadley et al., 2002).

However, standard passive training maneuvers or repetitive execution routines often fail in complete or severe incomplete SCI. This limitation is profoundly exacerbated by the physiological continuum of spinal shock, which suppresses sub-lesional network excitability across distinct chronological phases (Ditunno et al., 2004). Consequently, the volitional descending motor drive from the motor cortex is pathologically insufficient to re-activate dormant lumbosacral central pattern generators (CPGs). This gap in neurophysiological signaling underscores that standard rehabilitation lacks an active, intrinsic trigger to drive plasticity. This challenge represents a critical therapeutic bottleneck that can only be bridged by articulating physical training with active neuromodulation to lower the synaptic threshold required for functional circuit rewiring.

## 3 Rationale and design of multimodal synergistic therapy in SCI

**FIGURE 2:**
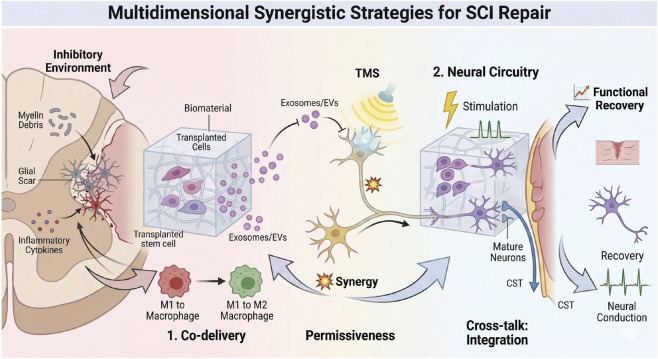
Schematic illustration of the multimodal synergistic therapy framework for spinal cord injury (SCI), highlighting the combinatory roles of stem cells, extracellular vesicles (EVs), biomaterials, and neuromodulation in remodeling the inhibitory microenvironment, promoting neural regeneration and circuit integration, and improving functional recovery. Abbreviations: SCI, spinal cord injury; EVs, extracellular vesicles.

SCI is a dynamic pathological process involving acute mechanical damage, secondary inflammation, oxidative stress, glial scar formation, demyelination, vascular dysfunction, and long-term failure of circuit reintegration. As a result, monotherapies often provide only partial benefit. An anti-inflammatory strategy may not restore tissue continuity, a scaffold may not provide sufficient biological instruction, and even axonal regrowth may not translate into functional recovery without effective circuit engagement. Thus, the rationale for multimodal therapy is not simply to combine treatments, but to integrate mechanistically complementary interventions in a stage-appropriate manner (Figure 2).

This framework can be understood through four major therapeutic modules. Stem cells act as biological effectors through paracrine signaling, immunomodulation, trophic support, and partial cell replacement. Extracellular vesicles extend these functions in a cell-free format and offer greater flexibility for molecular delivery. Biomaterials provide structural support, improve local retention, guide axonal growth, and enable controlled delivery of therapeutic cargo. Neuromodulation addresses a key limitation of regenerative repair by promoting circuit activation, synaptic integration, and activity-dependent plasticity. Synergy arises when these modules are designed as interdependent components rather than isolated additions.

A rational multimodal strategy should follow three principles. First, pathology matching: different interventions should be aligned with the dominant barriers present at acute, subacute, and chronic stages of SCI. Second, spatiotemporal coordination: therapeutic inputs should be delivered according to the evolving biology of the lesion rather than applied simultaneously without distinction. Third, functional integration: the aim is not to accumulate interventions, but to establish a coherent repair sequence linking microenvironment modulation, structural reconstruction, and circuit reactivation.

Importantly, multimodal design must also remain translationally feasible. Although increasing complexity may improve mechanistic coverage, it also raises challenges in manufacturing, standardization, safety evaluation, and regulatory approval. The most effective strategy for SCI is therefore unlikely to be the most complex one, but rather the one that best aligns pathological timing, biological rationale, engineering feasibility, and rehabilitation goals.

## 4 Stem cell therapy: a regenerative strategy driven by multiple synergistic mechanisms

In regenerative medicine for SCI, stem cell therapy has developed into a comprehensive therapeutic system characterized by synergistic mechanisms and integrated strategies (Sugai et al., 2025; Wang R. et al., 2025). Its core restorative principle has evolved beyond the early “cell replacement” paradigm toward a dominant focus on paracrine signaling-mediated microenvironmental remodeling (Shoemaker and Kornblum, 2016; Li Y. et al., 2023). Transplanted stem cells function as bioactive hubs that continuously secrete neurotrophic factors (e.g., BDNF, GDNF), immunomodulatory factors (e.g., IL-10, TGF-β, PGE_2_), and pro-angiogenic factors (e.g., VEGF). Through this secretion profile, they systematically inhibit neuroinflammation, support neuronal survival, promote angiogenesis, and modulate glial scar formation, collectively creating a permissive microenvironment conducive to axonal regeneration and neural circuit reorganization (Xiao and Czopka, 2023; Zaripova et al., 2023; Zhang et al., 2023; Zhang et al., 2024a; Zeng, 2025). Concurrently, a subset of stem cells retains differentiation potential and can directly differentiate into neurons or oligodendrocytes, thereby contributing to structural reconstruction.

At the fundamental research level, several breakthrough advances have been achieved, primarily reflected in the increased sophistication and diversification of intervention strategies. First, regarding translational models, a 2025 study demonstrated that human embryonic stem cell-derived spinal cord neural stem cells (H9-scNSCs) extensively restored forelimb function in rhesus macaques (Sinopoulou et al., 2025). These grafts overcame historical bottlenecks of low survival by matching normal spinal cord cell-fate proportions, completely filling massive lesion cavities, and extending hundreds of thousands of axons up to 39 mm to form functional synapses with host circuits (Sinopoulou et al., 2025). Second, the controlled activation of endogenous repair mechanisms has emerged as a new direction. Research indicates that the drug ampakine CX546 can effectively activate endogenous ependymal neural stem/progenitor cells (epNSPCs) by potentiating the α-amino-3-hydroxy-5-methyl-4-isoxazolepropionic acid receptor (AMPAR) signaling pathway, promoting their migration and integration, and thereby improving functional recovery (Hachem et al., 2025).

Recent transcriptomic analyses reveal that neural progenitor cell grafting sustains an embryonic-like “regenerative transcriptome” in injured neurons, centrally driven by the Huntingtin (Htt) gene. Building on this concept, a 2025 Nature study identified the approved drug thiorphan as a pharmacological mimic of this regenerative state. Co-delivery of thiorphan with neural stem cell (NSC) grafts produced a pharmaco-cellular synergy that significantly increased neurite outgrowth in aged human neurons and improved forelimb recovery by 50% in severe rat SCI models, establishing a new precision medicine paradigm (van Niekerk et al., 2025).

Furthermore, strategies for functionally modulating endogenous cells are continuously being refined. In oligodendrocyte precursor cell (OPC)-based therapies, beyond activating the cells themselves, optimizing the lesion microenvironment is also considered important for remyelination after SCI. This may involve reducing inhibitory extracellular cues and attenuating inflammatory signaling, although these mechanisms should be supported by SCI-specific studies (Xiao and Czopka, 2023). Genetic engineering and precision cell design constitute another active area of investigation. Wu et al. (Wu et al., 2024) engineered TrkC-modified, induced pluripotent stem cell (iPSC)-derived NSCs and combined them with a linearly ordered collagen scaffold loaded with the ligand Collagen-binding domain-neurotrophin-3 (CBD-NT3). In a complete spinal cord transection model, this approach not only ameliorated the injury microenvironment but also significantly promoted host axonal regeneration, synapse formation, and myelin repair, ultimately restoring motor function and electrophysiological conduction. Moreover, employing chemical reprogramming techniques, such as the specific cocktail LC, enables the direct conversion of endogenous reactive astrocytes into functional GABAergic neurons, offering a novel strategy for *in situ* repair (Tan et al., 2024). Concurrently, studies show that co-transplanting human iPSC-derived OPCs with human umbilical vein endothelial cells can improve neurological outcomes more effectively by harnessing the synergistic effects of promoting both angiogenesis and remyelination (Li et al., 2024).

Modifying the transplantation microenvironment is also critical for improving the therapeutic efficacy of iPSC-based therapies. Biomaterial-assisted hepatocyte growth factor (HGF) delivery has been shown to improve the local repair environment after SCI(Yamane et al., 2018). In addition, HGF-based combinational strategies may further enhance the therapeutic efficacy of hiPSC-derived neural stem/progenitor cell transplantation (Okano et al., 2025).

Clinical translation research is advancing steadily along multiple fronts, with accumulating data on early safety and preliminary efficacy. A first-in-human clinical study of hiPSC-derived neural stem/progenitor cell transplantation for subacute SCI has been initiated, with safety as the primary objective (Sugai et al., 2021). Progress has also been made in validating mature cell types. Phase I studies of autologous human Schwann cell (SC) transplantation in SCI have supported procedural feasibility and short-term safety, whereas evidence for efficacy remains preliminary (Sugai et al., 2021). Meanwhile, mesenchymal stem cell (MSCs) transplantation has demonstrated potential to improve neurological function in some acute and subacute patients, and a meta-analysis suggests a positive impact on outcomes for those with complete injuries (Liu Y. et al., 2025). MSC transplantation has shown therapeutic potential across preclinical and early clinical studies of SCI. Reviews have summarized possible benefits of both systemic and local MSC-based approaches, although specific claims regarding lesion homing or individual case-level sensory improvement should be supported by the original reports (Hirota et al., 2023; Xia et al., 2023).

Specifically, during the subacute phase when neural plasticity remains intact, treatment primarily focuses on inhibiting secondary damage and modulating the inflammatory microenvironment, often utilizing MSCs derived from bone marrow or umbilical cord. Studies indicate that 30.9% of patients achieve improvement in sensory function, with some showing an upgrade in the American Spinal Injury Association (ASIA) Impairment Scale from grade A to grade B or higher (Agosti et al., 2024). This suggests that the strategy helps stabilize homeostasis and awaken residual neural function (Khaing et al., 2023; Agosti et al., 2024). In the chronic phase, where the environment for regeneration is more challenging, the strategy shifts toward actively promoting neural regeneration and structural remodeling, explored via methods such as intraspinal injection or combination therapies. Although motor recovery in this context remains modest, these findings demonstrate the potential feasibility of late-stage intervention (Mu et al., 2024).

Multiple clinical trials have shown that stem cell therapies from various sources exhibit favorable safety profiles and potential efficacy in SCI treatment. Research on bone marrow-derived MSCs reported that the main adverse events were mild fever and headache, with no serious fatal incidents; a trial of allogeneic MSCs for chronic SCI further confirmed their safety. Regarding neural progenitor cells (NPCs), oligodendrocyte precursor cells derived from human embryonic stem cells (AST-OPC1) showed a good safety profile and partial improvement in functional scores in a Phase one/2a trial for subacute cervical SCI(Roman et al., 2024). Additionally, studies on umbilical cord-derived MSCs also suggest a positive role in promoting neural regeneration. Overall, current early-phase clinical evidence suggests that several stem cell-based approaches are feasible and appear reasonably safe, whereas their efficacy remains heterogeneous and requires validation in larger controlled trials (Table 1).

**TABLE 1:** Characteristics and applications of stem cells from different sources for SCI treatment.

Stem cell type	Primary source	Key paracrine factors	Major functions	Suitable injury stage	Representative clinical trial (NCT number)	Remarks
BM-MSCs	Bone marrow	VEGF, BDNF, IL-10, TGF-β	Immunomodulation, angiogenesis, neurotrophic support	Acute/Subacute	NCT02981576	Good safety profile; sensory improvement in some patients
UC-MSCs	Umbilical cord blood/Tissue	Similar to BM-MSCs, high VEGF expression	Anti-inflammatory, pro-angiogenic, supports neuronal survival	Acute/Subacute	NCT02481440	Abundant source, strong proliferative capacity
NSCs	Embryonic/Adult brain or spinal cord	BDNF, GDNF, NGF	Differentiation into neurons/glia; provides neurotrophic support	Chronic	Multiple early-stage trials	Direct neural replacement potential, but source limited
iPSC-NS/PCs	Somatic cell reprogramming	Can be engineered as needed	Personalized therapy; can differentiate into various neural cells	Subacute/Chronic	Early clinical translation is emerging	Avoids ethical issues, but requires tumorigenicity control
SCs	Peripheral nerve	Ngf, BDNF, GDNF, PMP22	Promotes remyelination; guides axonal growth	Chronic	NCT02354625 (autologous transplantation)	Phase I safety has been reported; efficacy signals remain preliminary
OPCs	Embryonic stem cells/Progenitor cells	PDGF-AA, FGF2	Remyelination; supports neuronal survival	Subacute/Chronic	AST-OPC1 trial (phase 1/2a)	Targets demyelinating injury

In summary, stem cell therapy for SCI has evolved into a comprehensive translational continuum—spanning molecular mechanism elucidation, engineered cell product development, and innovative clinical protocol design. The central challenges and future directions now lie in achieving a deeper convergence of personalized cell preparations, intelligent biomaterials, and precision rehabilitation strategies. Through rigorously conducted large-scale randomized controlled trials, these promising laboratory advances must ultimately be translated into clinically viable and accessible treatments with well-defined efficacy benchmarks. While stem cell therapy offers robust paracrine and regenerative potential, challenges such as low post-transplantation survival, potential tumorigenicity, and immune rejection remain significant hurdles. To circumvent these limitations, researchers have increasingly focused on the primary functional mediators of stem cells—EVs. EVs provide a relatively safer and more stable alternative that preserves many of the microenvironmental remodeling functions of their parent cells, thereby representing an important conceptual progression within the landscape of biological therapies for SCI.

## 5 Extracellular vesicles: a multifunctional communication platform mediating microenvironmental remodeling and neural repair

In SCI regenerative medicine, EVs have emerged as crucial mediators of intercellular communication with multifaceted reparative potential that extends far beyond their role as mere drug delivery vehicles. They are increasingly recognized as active regulators of neuroinflammation, vascular remodeling, and neural repair (Mushtaq et al., 2024). Derived from diverse cellular sources, EVs carry a variety of bioactive components—including proteins, microRNAs, and lipids—that enable them to precisely regulate neuroinflammation, promote neural regeneration and remyelination, inhibit multiple forms of cell death, and enhance angiogenesis and blood-spinal cord barrier repair. Collectively, these actions help shape a microenvironment that supports neural structural rebuilding and functional recovery (Liu and Chen, 2019).

### 5.1 Progress in mechanistic research

At the fundamental research level, therapeutic strategies involving EVs are advancing rapidly toward greater sophistication. In terms of mechanistic insights, studies have moved beyond simply confirming that EVs alleviate neuroinflammation by delivering specific miRNAs to modulate microglia/macrophage polarization. Recent research revealed that miRNA let-7b-5p, delivered by NSC-derived EVs, can target and suppress LRIG3 expression, thereby mitigating macrophage pyroptosis (Liu et al., 2024a). The role of EVs in inhibiting cell death through the regulation of multiple signaling pathways has also been elucidated. For instance, Qin et al. (Qin et al., 2024) found that exosomes from epidermal growth factor receptor-positive neural stem cells (EGFR^+^ NSCs-Exos) are enriched with miR-34a-5p, which promotes axonal regeneration by inhibiting Histone deacetylase 6 (HDAC6) expression—a mechanism that stabilizes microtubules and activates autophagy. Separately, NSC-derived exosomes have been shown to inhibit neuronal necroptosis by disrupting the interaction between receptor-interacting serine/threonine-protein kinase 1 (RIPK1) and receptor-interacting serine/threonine-protein kinase 3 (RIPK3) (Li S. et al., 2025), while MSC-derived exosomes have also been reported to suppress neuronal apoptosis through modulation of the PTEN/AKT/mTOR signaling axis (Liu B. et al., 2022).

Regarding functional enhancement, EVs can deliver specific factors such as VGF nerve growth factor inducible (VGF) and miR-133 b to promote axonal regeneration and myelination (Li et al., 2018). Specifically, VGF carried by MSC-derived exosomes enhances oligodendrogenesis, and their miR-133 b content promotes axonal regeneration by activating the ERK1/2-STAT3-CREB pathway (He et al., 2022). Furthermore, SC-derived exosomes have been shown to promote axonal growth after SCI by decreasing protein tyrosine phosphatase-sigma (PTP-σ) activation on chondroitin sulfate proteoglycans (CSPGs) via the Rho/rho-associated protein kinase (ROCK) pathway (Zhu et al., 2024). Expanding beyond MSC-, NSC-, and peripheral glia-derived EVs, endogenous central nervous system glial cells may also contribute to regenerative repair. Notably, NG2 glia-derived exosomes have been demonstrated to promote neural regeneration, remyelination, and functional recovery after injury (Goncalves et al., 2018; Goncalves et al., 2019).

### 5.2 Engineered modifications and synergistic therapies

Engineering modification is a key strategy for enhancing the targeted therapeutic efficacy of EVs. Conventional pharmacotherapies are heavily bottlenecked by the blood-spinal cord barrier (BSCB), resulting in low drug bioavailability at the lesion and severe systemic side effects. To overcome this mode-of-administration challenge, researchers have developed diverse peptide modification strategies to improve their *in vivo* delivery. Functionalizing EVs with targeting moieties (e.g., CAQK or TAT peptides) allows these cell-free vehicles to cross the BSCB actively, shifting the paradigm from systemic toxic exposure to localized, precision-targeted molecular regulation. For instance, EVs derived from human umbilical cord MSCs, when functionalized with TAT and RVG peptides, can efficiently encapsulate GPX4 activators, cross the blood-spinal cord barrier, and localize to the injury site, ultimately activating GPX4 to inhibit ferroptosis by mitigating lipid peroxidation and rescuing the sub-organelle defense mechanism within vulnerable neurons (Zhou et al., 2024; Fan et al., 2025). Similarly, CAQK-modified extracellular vesicles loaded with C-C Motif Chemokine Ligand 2 (CCL2)-siRNA enable targeted delivery to the SCI region and suppress local inflammatory signaling, thereby promoting repair (Liu and Chen, 2019; Rong et al., 2023).

Pretreatment strategies have also been employed to optimize EV function. EVs released by TGF-β1-preconditioned mesenchymal stem cells promote recovery after SCI, in part by enhancing endogenous NSC proliferation and modulating neuroinflammation (Chen et al., 2024). Furthermore, multidimensional engineering overcomes natural EV limitations. A 2025 study developed C-A/R-EVs, combining Angiopep-2 and RGD peptides to cross the blood-spinal cord barrier and target neovasculature. Derived from curcumin-pretreated cells, these anti-inflammatory EVs precisely reprogram microglia toward a reparative phenotype—confirmed via single-nucleus RNA sequencing—accelerating debris clearance and barrier restoration (van Niekerk et al., 2025). This transcriptomic shift directly correlates with recent single-cell RNA sequencing and spatial transcriptomic datasets showing that targeted EV administration systematically reshapes myeloid cell heterogeneity, suppressing the disease-associated microglia (DAM) transition and alleviating necroinflammation within the degenerative niche (Zhang et al., 2026).

**FIGURE 3:**
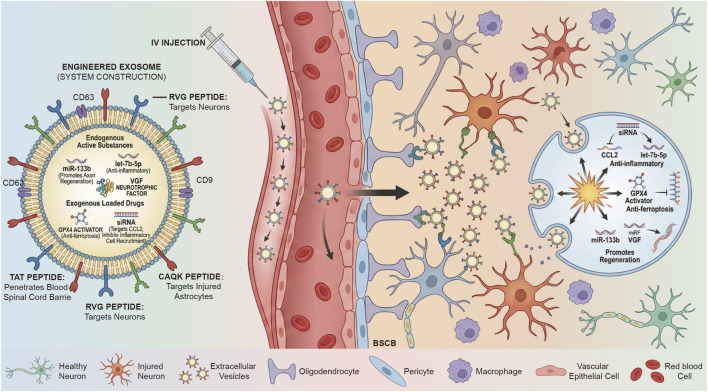
Schematic illustration of engineered extracellular vesicles (EVs) as a targeted drug delivery system for spinal cord injury (SCI) repair. EVs can be functionalized with targeting peptides (e.g., CAQK, RVG, TAT), loaded with therapeutic cargos such as GPX4 activators, siRNAs, or neurotrophic factors, and designed to cross the blood-spinal cord barrier (BSCB). Upon reaching the injury site, EVs modulate the local microenvironment by reducing ferroptosis, neuroinflammation, and glial scarring, while promoting axonal regeneration and remyelination. Abbreviations: EV, extracellular vesicle; SCI, spinal cord injury; BSCB, blood-spinal cord barrier; GPX4, glutathione peroxidase 4; siRNA, small interfering RNA.

Combination therapies further demonstrate synergistic benefits. An injectable Decellularized extracellular matrix (dECM) hydrogel loaded with exosomes secreted by cortical neurons derived from human iPSCs enhanced tissue repair following traumatic SCI(Wang et al., 2024). In another approach, Li et al. (Li Y. et al., 2025) combined human iPSC-derived spinal cord organoids with gelatin methacryloyl (GelMA) hydrogel for transplantation, which effectively enhanced neuronal integration while reducing glial scarring and neuroinflammation. Crucially, when incorporated into functional bio-scaffolds or paired with inorganic nanozymes, these smart EV-biomaterial hybrid systems offer an expansive biophysical framework capable of simultaneously restoring mitochondrial quality control (MQC), balancing copper-induced proteotoxic stress, and intercepting alternative cell death pathways like cuproptosis and ferroptosis, bridging the gap between passive structural gap-filling and active metabolic homeostasis (Wang Q. et al., 2025; Li et al., 2026). A representative schematic of such engineered exosome platforms is illustrated in Figure 3, which depicts the key design features, including targeting peptide modification, cargo loading, and blood–spinal cord barrier penetration, that enable precise delivery and microenvironmental modulation after SCI.

### 5.3 Current status and challenges in clinical translation

Regarding clinical translation, EV-based therapy remains largely in an early exploratory phase, though its development trajectory is becoming clearer. While large-scale randomized controlled trials are currently lacking, the inherent advantages of EVs—such as low immunogenicity, good biocompatibility, high stability, and the ability to easily cross the blood–spinal cord barrier—render their clinical translation highly promising (Mao et al., 2025).

Clinically, EV therapy faces a “heterogeneity black box” where conventional extraction cannot separate highly active from inert vesicles. Overcoming this requires microfluidics for high-purity isolation and CRISPR-Cas9 genome editing of parent cells for active, targeted cargo loading, shifting the paradigm from passive extraction to precise therapeutic engineering (Wang et al., 2022). Nevertheless, several EV-based therapeutic strategies are gradually moving toward clinical application. Future efforts aimed at optimizing manufacturing processes, identifying precise key active molecules, and developing efficient delivery systems are expected to steadily advance EVs into early-stage human clinical studies (Table 2). However, despite the immense biological potential of both stem cells and EVs, their standalone application often suffers from rapid clearance and diffusion away from the injury site. Furthermore, because most SCI cases are traumatic in origin, severe injuries commonly involve tissue disruption, hemorrhage, myelin debris accumulation, and cavity formation, creating a physical defect that cannot be bridged by biological factors alone (Mensah et al., 2025). In addition, chronic posttraumatic syringomyelia may develop slowly over years or even decades, resulting in progressive cavitation and neurological deterioration. These clinical realities indicate that biomaterial-based interventions must be considered not only as regenerative scaffolds, but also in relation to feasible routes of administration, including intraoperative implantation during decompression or later invasive delivery in selected chronic cases. Therefore, to maximize local retention of therapeutic agents and provide a structural substrate for axonal regrowth, the integration of biomaterials becomes indispensable. Engineered scaffolds and targeted delivery systems not only enable spatiotemporal control over drug and vesicle release but also physically reconstruct the neural matrix, thereby laying the foundation for multimodal synergistic therapy.

**TABLE 2:** Key active molecules carried by extracellular vesicles and their functions.

Type of active molecule	Specific molecule	Source of EVs	Target gene/Pathway	Downstream biological effect
miRNA	miR-133 b	MSC-derived exosomes	ERK1/2-STAT3-CREB	Promotes axonal regeneration and functional recovery
miRNA	Let-7b-5p	NSC-derived exosomes	LRIG3	Inhibits macrophage pyroptosis, alleviates neuroinflammation
miRNA	miR-34a-5p	EGFR^+^ NSC-derived exosomes	HDAC6	Stabilizes microtubules, promotes autophagy and axonal regeneration
miRNA	miR-26a	MSC-derived exosomes	PTEN/AKT/mTOR	Inhibits neuronal apoptosis
Protein	VGF	MSC-derived exosomes	Not specified	Promotes oligodendrogenesis, enhances myelin repair
Functional protein	GPX4 activator	Engineered HUCMSC-derived EVs	GPX4	Inhibits ferroptosis, protects neurons
siRNA	CCL2-siRNA	CAQK-peptide modified EVs	CCL2 gene	Silences local inflammatory factor expression, reduces scar formation

From a translational and bioengineering lens, transitioning these sophisticated cell-seeded or EV-loaded scaffolds from bench to bedside introduces formidable regulatory and industrial manufacturing hurdles. When biological components are integrated with synthetic or natural polymer matrices, the resulting constructs are classified as combination products under major global regulatory frameworks (Sekar et al., 2021; Gupta et al., 2024). Under the U.S. FDA framework, the assignment of a premarket review pathway is stringently dictated by the product’s Primary Mode of Action (PMOA), typically designated by the Office of Combination Products (OCP) (Belsky and Smiell, 2021; Gupta et al., 2024). If the therapeutic effect is primarily driven by the living cellular or bioactive EV constituent, the construct is governed by the Center for Biologics Evaluation and Research (CBER) via the rigorous Biologics License Application (BLA) pathway, while concurrently requiring the Center for Devices and Radiological Health (CDRH) to evaluate the structural scaffold component (Proffen et al., 2015; Belsky and Smiell, 2021). Similarly, under the National Medical Products Administration (NMPA) framework in China, complex regenerative constructs cannot rely on simple substantial equivalence to historical acellular device predicates. Instead, these biomimetic scaffolds exhibiting active tissue remodeling or biological therapeutic claims are subjected to comprehensive joint technical reviews, strictly requiring exhaustive prospective clinical trial data to navigate regulatory approval (Liu et al., 2021).

Compounding these regulatory pathways are the pronounced engineering and manufacturing bottlenecks associated with scaling up these therapeutics under current Good Manufacturing Practices (cGMP). Unlike traditional synthetic small molecules or well-characterized acellular matrices, the scale-up of engineered exosomes suffers from inherently complex Chemistry, Manufacturing, and Controls (CMC) challenges (Gupta et al., 2024). Culturing parent stem cells at an industrial scale often introduces genetic and phenotypic drift, which directly compromises batch-to-batch consistency and the stability of critical quality attributes (CQAs) of the harvested EVs(Lee et al., 2014). Furthermore, a significant barrier to commercialization is the lack of decentralized standardization in analytical characterization methodologies across the nanomedicine and extracellular vesicle landscape. Without standardized, high-throughput reporting systems to quantify exosomal isolation purity, transmembrane targeted-peptide presentation, and precise intra-vesicular cargo loading efficiency, ensuring the clinical reproducibility and long-term potency of engineered EV-scaffold combinations remains a central bottleneck in the pipeline of regenerative medicine (Sharifi et al., 2022).

## 6 Biomaterials: engineered scaffolds and delivery systems for structural support and spatiotemporally controlled therapy

While standard surgical decompression effectively relieves mechanical pressure on the injured cord, it typically leaves a physical cavity and a chronically hostile lesion site. Advancements in biomaterial engineering directly leverage this surgical access; instead of a simple closure, the intraoperative window provides a unique opportunity for the *in situ* administration of smart hydrogels and aligned scaffolds. These materials physically bridge the lesion volume and reconstruct the neural matrix, transforming a standard decompression surgery into a highly localized, collaborative therapeutic platform.

### 6.1 Inorganic nanomaterials

Gold nanoparticles (AuNPs), selenium nanoparticles (SeNPs), ceria nanoparticles (CeONPs), zinc oxide nanoparticles (ZnO NPs) and magnetic nanoparticles (MNPs) have all been investigated as potential nanomaterial platforms for SCI repair (Zare et al., 2022; Fleming et al., 2023; Gong et al., 2023; Yang Q. et al., 2025). Despite their distinct chemical properties, these materials share key common features—including biocompatibility, antioxidant and anti-inflammatory activities, anti-apoptotic effects, and surface modifiability—which collectively provide a foundation for their application in neural repair (Yang Q. et al., 2025).

These materials generally exhibit excellent biocompatibility, remaining stable *in vivo* without inducing significant cytotoxicity or immune rejection, which makes them suitable for long-term implantation or local delivery. A core biological function shared by many is the efficient scavenging of reactive oxygen species (ROS). For instance, certain nanomaterials can continuously suppress oxidative stress by mimicking the activity of endogenous antioxidants such as superoxide dismutase (SOD). AuNPs possess notable antioxidative properties, directly neutralizing ROS and restoring levels of endogenous antioxidants including SOD, thereby protecting mitochondrial function and mitigating oxidative damage to vulnerable neural tissue (Chiang and Nicol, 2022; Jiang et al., 2022). Similarly, CeONPs leverage the reversible Ce^3+^/Ce^4+^ redox cycle and abundant surface oxygen vacancies to alleviate oxidative stress at the injury site, scavenge ROS, shield neurons from further damage, and foster a microenvironment conducive to neural repair and regeneration (Hosseini and Mozafari, 2020; Wei et al., 2021).

In terms of immunomodulation, these nanomaterials can effectively modulate the activation states of microglia and astrocytes, downregulate the expression of pro-inflammatory factors such as TNF-α and IL-1β, and promote the secretion of IL-10. Collectively, these actions drive the local immune microenvironment toward an anti-inflammatory, pro-repair phenotype. Their anti-apoptotic effects are primarily mediated by regulating the expression of Bcl-2 family proteins and inhibiting the caspase cascade, thereby reducing programmed cell death in neurons and glial cells and preserving the structural integrity of neural tissue in the injured area (Chiang et al., 2020; Chiang et al., 2024).

The surfaces of these materials are amenable to chemical modification, offering considerable potential for functionalization. Polyethylene glycol (PEG) modification can prolong their *in vivo* circulation time, while hyaluronic acid (HA) modification leverages the high expression of the CD44 receptor at the injury site to achieve targeted enrichment and improve therapeutic precision. Certain functionalized systems can further influence the differentiation fate of NSCs, promoting their commitment to neuronal lineages while suppressing aberrant astrocyte proliferation, thereby favoring synaptic remodeling and neural circuit recovery (Salathia et al., 2023).

In terms of application, these inorganic nanomaterials are often integrated with biological carriers such as hydrogels or nanofiber scaffolds to form multifunctional composite systems. Such integrated structures combine sustained drug release, physical support, and microenvironment modulation. For example, a piezoelectric catalytic system composed of AuNPs and barium titanate can continuously release hydrogen under mechanical stimulation, enabling *in situ* scavenging of ROS(You et al., 2024). Gold nanoparticle-based systems have shown potential in SCI-related scaffold engineering, for example, by supporting neuronal differentiation and functional connection formation (Ko et al., 2022).

SeNPs modified with ultra-small lentinan exhibit a particle size of approximately 10 nm, enabling penetration of the blood–spinal cord barrier. They can also synergize with L-arginine to enhance the expression of antioxidant selenoproteins (Liu P. et al., 2025). SeNPs co-modified with tanshinone IIA (or ligustrazine) and astragalus polysaccharide demonstrate the advantages of multi-component synergistic regulation (Rao et al., 2022; Mai et al., 2025). Additionally, a hydrogel based on oxidized sodium alginate and N-succinyl chitosan loaded with selenium–folic acid nanoparticles possesses antioxidant, antibacterial, and controlled-release properties (Farzan et al., 2025). CeONPs mimic the activities of multiple antioxidant enzymes via reversible Ce^3+^/Ce^4+^ valence-state transitions (Behroozi et al., 2022). The AhCeO_2_-Gel hydrogel promotes the differentiation of NSCs into neurons and induces microglial polarization toward the M2 phenotype through nitric oxide (NO) release (Liu D. et al., 2025). When incorporated into a gelatin-polycaprolactone scaffold, it provides structural guidance for regenerating axons and helps inhibit cystic cavity expansion (Rahimi et al., 2023).

Additionally, zinc oxide nanoparticles (ZnO NPs) have been explored for their unique neuroprotective potential. Utilizing an *in vitro* microfluidic neuronal axotomy model, safe doses of ZnO NPs (5 μg/mL) were shown to significantly suppress oxidative stress and neuronal apoptosis by upregulating SOD1/2 expression and restoring mitochondrial membrane potential. Transcriptomic analyses indicate that these anti-inflammatory and antioxidative effects are predominantly mediated by modulating the PI3K/Akt signaling pathway, thereby promoting axon regeneration (Liu J. et al., 2022).

MNPs possess unique physiologically responsive properties that allow targeted delivery of neurotrophic factors or cells under an external magnetic field (Zhuo et al., 2024; Senanayake et al., 2025). Under an alternating magnetic field, they can generate thermal effects or magneto-mechanical forces that physically disrupt glial-scar structure and modulate cell-membrane signaling (Du et al., 2025). When combined with biomaterials, MNPs form magnetically active systems capable of directing cell migration and axonal growth *in vitro* under magnetic guidance (Hu et al., 2021). ROS-responsive mesoporous-silica-loaded hydrogels have been developed for SCI repair and can promote axonal regeneration and functional recovery by modulating injury-associated signaling pathways (Zhang K. et al., 2025).

All studies to date have been conducted *in vitro* or in animal models. While certain materials have demonstrated promising outcomes in animal experiments—such as improved motor function and reduced lesion volume—none have advanced to clinical trials. The available data remain entirely preclinical, with critical gaps in long-term toxicity, *in vivo* pharmacokinetics, and cross-species validation. Consequently, clinical translation remains a considerable challenge.

### 6.2 Organic polymeric nanomaterials

In SCI regenerative medicine, organic polymer nanomaterials have emerged as important therapeutic platforms with unique advantages and multifaceted reparative potential. Moving beyond the conventional role of drug delivery vehicles, these materials actively modulate the injured microenvironment, promote neural regeneration and remyelination, inhibit cell death, and enhance angiogenesis and blood-spinal cord barrier repair. Through these combined actions, they establish a favorable milieu for structural reconstruction and functional recovery of neural tissue.

In recent years, polymeric micelle and nanosphere systems have demonstrated considerable promise for SCI treatment. For instance, sialic acid-modified PEG-PLGA micelles enable precise targeting and delivery of minocycline by specifically recognizing E-selectin expressed on damaged endothelial cells (Wang et al., 2019). Polysialic acid-based micellar systems offer dual functionality: in addition to serving as drug carriers, their intrinsic material composition actively promotes axonal outgrowth and remyelination, thereby providing direct support for neural repair. Furthermore, CAQK-peptide-modified nanosystems represent a breakthrough in targeted delivery, showing specific accumulation within scar tissue at the injury site and enhancing therapeutic outcomes (Wang et al., 2020). Collectively, these systems constitute a multi-layered interventional strategy tailored to the complex microenvironment of SCI.

Lipid-based carrier systems demonstrate distinct advantages in the treatment of SCI. Conventional liposomes and solid lipid nanoparticles (SLNs) have been successfully employed to deliver inhibitors of macrophage migration inhibitory factor (Saxena et al., 2015). Of particular interest are SLN systems engineered with a high density of thioether moieties, which not only deliver therapeutic agents but also directly scavenge excess ROS at the lesion site, thereby exerting anti-inflammatory and anti-apoptotic effects (Iannotti et al., 2011). Lipid nanoparticle (LNP)-mediated mRNA delivery represents a novel direction in gene therapy, enabling the *in situ* expression of various therapeutic proteins directly within the injured tissue (Moosavi et al., 2025). In terms of targeting strategy, the dual-targeting liposomal system (bFGF@Lip-Cp&Rp) integrates both the CAQK peptide for lesion-specific targeting and the R_2_KC peptide for enhanced penetration across the blood–spinal cord barrier, significantly improving delivery efficiency (Wu et al., 2023). Furthermore, the MP-LNP system innovatively replaces conventional cholesterol with myelin basic protein (MP) to encapsulate C3 transferase mRNA, achieving co-delivery of anti-inflammatory agents and neural repair genes, which offers a novel therapeutic strategy for SCI(Dong et al., 2025).

Dendritic polymer systems, such as the CMCht/PAMAM complex, have gained attention in SCI treatment owing to their well-defined branched architecture and high drug-loading capacity. These dendritic systems can sustain drug release for up to 14 days, supporting long-term therapeutic regimens (Cerqueira et al., 2013; Yu et al., 2022). Polyelectrolyte complex nanoparticles, particularly those based on chitosan, offer a protective microenvironment for protein-based therapeutics, preventing their inactivation during delivery. The superoxide dismutase “nanozyme” technology, which employs a multilayer electrostatic encapsulation strategy, successfully preserves long-term enzymatic activity, providing an effective means to continuously scavenge excess ROS at the injury site (Nukolova et al., 2018; Xu et al., 2019). Collectively, these innovative systems expand the scope of polymeric nanomaterials in SCI therapy.

Composite delivery systems, which integrate the advantages of diverse materials, represent a cutting-edge direction in SCI therapy. In hydrogel-nanoparticle composite systems, an injectable *in situ* gel composed of carboxymethyl cellulose sodium and chitosan and loaded with cannabidiol combines the sustained local retention of hydrogels with the deep tissue penetration of nanoparticles, achieving more comprehensive therapeutic coverage (Zhang et al., 2022). A dual-layer construct featuring an outer chitosan hydrogel and an inner core of mesoporous silica nanospheres enables the spatiotemporally sequential release of different drugs, precisely aligning with the biological timeline of SCI repair. By first releasing anti-inflammatory agents to suppress acute-phase injury and subsequently delivering neurotrophic factors to promote regeneration, such systems achieve optimized temporal control of the therapeutic process. Together, these composite platforms provide an integrated strategy to address the multistage, multitarget challenges inherent in SCI treatment.

Although large-scale randomized controlled clinical trials are still lacking, organic polymer nanomaterials show promising clinical translation potential due to their low immunogenicity, favorable biocompatibility, and ability to cross the spinal cord barrier. Current challenges primarily include the absence of standardized preparation and quality control systems, unclear *in vivo* pharmacokinetic profiles and long-term safety, suboptimal targeting efficiency, significant material heterogeneity, and difficulties in scaling up production. Nevertheless, with ongoing technological advances and process optimization, several therapeutic strategies based on these materials are gradually progressing toward clinical application. Looking ahead, the establishment of unified standards, identification of key active ingredients, and development of efficient delivery systems are expected to facilitate their entry into early-stage clinical research.

### 6.3 Natural biomaterials

Natural biomaterials hold significant value in tissue engineering and regenerative medicine. Typically characterized by excellent biocompatibility, controllable degradation, and high cell affinity, these materials can mimic the extracellular matrix microenvironment. They provide a temporary scaffold for tissue repair and actively promote regeneration. Sourced from widely available and renewable origins, their degradation products are generally non-toxic and can be metabolized by the body. These materials inherently contain bioactive groups that support cell adhesion, proliferation, and differentiation, facilitating favorable interactions with host tissues. Furthermore, their inherent softness and ease of processing make them particularly suitable for forming hydrogels to fill irregular defects. They also serve effectively as carriers for drugs, growth factors, and cells, enabling collaborative repair strategies.

Research on natural biomaterials in the field of SCI repair is steadily expanding. Among protein-based materials, collagen—a core component of the extracellular matrix—exhibits high affinity for cell-surface integrins via its triple-helix structure, effectively promoting cell adhesion and growth (Bächinger and Boudko, 2025). Studies show that collagen scaffolds loaded with paclitaxel liposomes can activate the Wnt/β-catenin signaling pathway and guide the differentiation of NSCs into neurons (Li Z. et al., 2023). Gelatin, a hydrolyzed derivative of collagen, exhibits lower antigenicity. Its methacrylic-anhydride-modified photosensitive derivative, GelMA allows tunable mechanical properties and, when loaded with stem cells, reduces scar formation and promotes motor function recovery. More notably, conductive GelMA hydrogels combined with electrical stimulation can promote the directional adhesion and differentiation of neural cells. Furthermore, GelMA-based hydrogels can serve as local delivery depots for bioactive vesicles; for example, exosome-loaded GelMA-containing patch systems have been reported to promote neural regeneration after SCI (Qin et al., 2024). Separately, aligned conductive Carbon nanotube/hyaluronic acid methacryloyl (CNT/GelMA) hydrogel fibers combined with electrical stimulation further enhance directional neural growth and functional recovery (Yao et al., 2024). In addition, gelatin methacryloyl/hyaluronic acid methacryloyl (GelMA/HAMA) hydrogels constructed by 3D bioprinting technology, encapsulating NSC-derived living neural-like fibers, significantly enhance functional recovery through a triple mechanism involving immunomodulation, angiogenesis, and axonal guidance (Yang et al., 2023). Another line of research has developed dual-cross-linked biomimetic composite hydrogels that combine acellular spinal cord matrix (ASCM) with gelatin derivatives, incorporate Wnt agonists, and are reinforced with 3D-printed aligned microfibers. These constructs enable sustained drug release and promote the migration and differentiation of endogenous NSCs (Zhao et al., 2023).

Decellularized scaffolds preserve the natural extracellular matrix composition and three-dimensional network architecture, exhibit very low immunogenicity, and can support stem cells in maintaining stemness while promoting long-term nerve fiber regeneration. Notably, neonatal acellular spinal cord matrix enriched with pro-neurodevelopmental proteins has been shown to more effectively promote the proliferation, migration, and differentiation of NPCs (Sun et al., 2024). A carboxymethyl cellulose/quaternized chitosan hydrogel loaded with polydopamine nanoparticles promoted SCI recovery through anti-ferroptosis effects and modulation of macrophage polarization (Shi et al., 2024). The stiffness of alginate hydrogels significantly influences host response and axonal regeneration, with softer scaffolds reducing foreign-body reactions and limiting glial activation (Babaei et al., 2023). Meanwhile, biomimetic multi-channel silk fibroin nerve conduits have shown the ability to enhance SCs migration and drive macrophage polarization toward the M2 phenotype, effectively inhibiting glial scar formation and promoting nerve regeneration (Yuan et al., 2024).

Current research is advancing toward greater integration and functionalization. Scientists are enhancing material performance through chemical modification, constructing composite scaffolds that combine bioactivity with mechanical support, and developing multifunctional materials with aligned structures, conductivity, or sustained drug release. The introduction of advanced manufacturing technologies, such as 3D printing, now enables precise fabrication of these materials, offering new therapeutic promise for refractory conditions like SCI. These developments indicate that rationally engineered natural biomaterials hold the potential to overcome the limitations of conventional treatments and provide more effective solutions for neural tissue repair.

### 6.4 Synthetic biomaterials

Synthetic biomaterials exhibit distinct advantages compared to their natural counterparts. Their ease of industrial-scale production, along with highly tunable physicochemical properties—such as mechanical strength, degradation rate, and porosity—makes them particularly suitable for constructing long-lasting support structures and precise drug delivery systems. Through engineered design, these materials can be tailored to meet specific biomedical requirements, offering important technical support for SCI repair. However, synthetic biomaterials also share common limitations, including a lack of intrinsic bio-recognition sites and the potential for acidic degradation products to trigger inflammatory responses. These challenges have motivated researchers to integrate the physical advantages of synthetic materials with natural components or bioactive molecules via composite strategies, thereby constructing ideal repair scaffolds that combine mechanical support with biological functionality.

Regarding research advances, significant breakthroughs have been made in synthetic peptide-based materials. Self-assembling peptides composed of natural amino acids can form extracellular matrix (ECM)-like nanofiber networks *in vivo*, providing a three-dimensional physical scaffold and essential biochemical cues for axonal regeneration. Studies have confirmed that such peptide-based scaffolds effectively promote axonal regrowth and improve neurological recovery after central nervous system injury (Ellis-Behnke et al., 2006). Owing to their favorable biocompatibility and tunable physicochemical properties, peptide hydrogels can mimic the topography of the neural matrix and support neuronal cell adhesion, differentiation, and axon elongation (Xie et al., 2024). In 2023, a hybrid self-assembling gel combining synthetic peptides and silk fibroin was reported to effectively promote nerve regeneration and functional recovery in rats with SCI (Feng et al., 2023). Also in the same year, a natural peptide termed VD11 was shown to directly enhance neuronal survival and axonal outgrowth by alleviating oxidative stress and inflammation (Li S. S. et al., 2023). Most recently, in 2025, a 3D-bioprinted dynamic scaffold integrating BDNF with an N-cadherin-mimetic peptide was shown to guide rapid self-organization of neural networks and achieve marked repair in injury models (Yang J. et al., 2025). Together, these findings demonstrate that bioactive peptides and their composite systems offer a versatile, multifunctional strategy for spinal cord regeneration, underscoring the clinical translational potential of interdisciplinary research and development.

Polycaprolactone (PCL) has established itself as an important synthetic polymer in SCI tissue engineering due to its favorable biocompatibility, degradability, and ease of processing. Current research has expanded from merely using PCL-based materials to construct physical scaffolds for guiding axonal regeneration, to also developing novel PCL-based systems capable of actively modulating the injured microenvironment. For example, a composite scaffold assembled from PCL nanofibers and macrophage membranes can specifically adsorb and remove myelin debris from the lesion area, thereby inhibiting foam cell formation and neuroinflammation at the source. Preclinical studies have demonstrated its potential to reduce glial scarring and promote functional recovery (Zhou et al., 2025). Meanwhile, as a functional carrier, PCL is often combined with natural materials such as gelatin and loaded with active components like CeONPs to enhance antioxidant and neuroprotective effects. It continues to be explored as a platform for delivering NSCs or neurotrophic factors (Rahimi et al., 2023; Ríos et al., 2025). Overall, PCL occupies an important position in biomimetic neural scaffold design. Future studies must further evaluate its long-term safety and efficacy in large animal models to advance its clinical translation.

In the field of novel synthetic nanomaterials, oxygen-vacancy-deficient non-stoichiometric copper hydroxide loaded with electron-rich Ru clusters (Ru/def-Cu(OH)_2_) serves as an efficient biocatalytic ROS scavenger, inhibiting inflammatory cascades and remodeling a microenvironment conducive to regeneration (Liu et al., 2024b). Nanofiber guidance conduits fabricated via electrospinning and mask-coaxial electrospraying integrate multiple gradient cues—including topological, haptic, and chemotactic signals—to synergistically promote cell migration, neural differentiation, and axonal outgrowth, thereby regulating cellular homeostasis within the microenvironment (Zhang X. et al., 2025). Chiral nanoagonists targeting the epidermal growth factor receptor (EGFR) have been incorporated into biodegradable dressings; these agents activate downstream signaling pathways and promote neuronal behavior (Zhao et al., 2025). Recently, biomaterials have transitioned from structural scaffolds to active orchestrators of metabolic homeostasis. For example, a dual-cross-linked conductive hydrogel containing black phosphorus nanosheets (BP@Hydrogel) generates electrical signals under a magnetic field. Crucially, this bioelectric cue may help balance intracellular ion currents and activate the pro-survival PI3K/AKT pathway, thereby providing a plausible basis for maintaining mitochondrial homeostasis, preserving membrane potential, and reducing ROS generation that contributes to inflammatory regulated cell death (Liu M. et al., 2024). Furthermore, when coordinated with artificial biocatalytic nanozymes, including AhCeO2-Gel and electron-rich ruthenium-copper hydroxide clusters, these systems may establish an antioxidant shield that attenuates mitochondrial oxidative stress and helps preserve GPX4-related anti-ferroptotic activity. Thus, conductive hydrogel–nanozyme systems should be viewed not only as physical gap-filling scaffolds, but also as bioactive platforms with potential to modulate ferroptosis-, cuproptosis-, and PANoptosis-associated pathological cascades (Wang Q. et al., 2025; Li et al., 2026). These innovative studies provide diversified material-based strategies for SCI repair, highlighting the considerable potential and broad application prospects of synthetic biomaterials in neural regeneration.

Ultimately, while biological therapies and biomaterial scaffolds can successfully reconstruct the anatomical continuity and foster a pro-regenerative microenvironment, structural repair does not automatically translate to functional recovery. Regenerating axons must form meaningful synapses and integrate into active neural circuits. This crucial functional bridge is provided by neuromodulation technologies. By applying targeted electrical or magnetic stimulation, these techniques awaken dormant circuits and guide the functional integration of newly formed connections, representing the critical final puzzle piece in restoring volitional control.

## 7 Reconstructing neural circuits: neuromodulation and electrical stimulation for functional recovery

In recent years, advances in neuromodulation and electrical stimulation technologies have introduced additional strategies for functional recovery. By modulating neural circuit excitability and neural connectivity, these technologies are gradually moving from laboratory research toward clinical application. They offer therapeutic potential for patients, ranging from the recovery of standing and walking abilities to improvements in bowel and bladder function, suggesting a shift in SCI treatment from supportive rehabilitation toward more active neurological restoration strategies.

Among various technical approaches, spinal cord stimulation (SCS) has been the most extensively studied. Invasive epidural electrical stimulation (EES), which involves surgically implanted electrodes, enables direct modulation of spinal neural networks. Numerous clinical studies have confirmed that EES can effectively activate central pattern generators in the lumbosacral region, assisting patients with chronic complete SCI in regaining the ability to stand, step, and even perform limited over-ground walking (Harkema et al., 2011; Gill et al., 2018). Its application has expanded from lower-limb rehabilitation to upper-limb and hand-function recovery, and it has also demonstrated considerable potential in improving autonomic functions such as bladder control and sexual performance, highlighting its broad impact on multiple physiological systems (Moritz et al., 2024; Rybka et al., 2024). Complementing EES, non-invasive transcutaneous spinal cord stimulation (tcSCS) delivers current through the surface of the body to safely enhance spinal cord excitability. Owing to its non-invasive nature and ease of integration with conventional rehabilitation training, tcSCS has become a key research focus for promoting motor recovery in patients with incomplete SCI(Tajali et al., 2024).

Beyond interventions directly targeting the spinal cord, brain stimulation techniques have opened another important avenue for promoting plasticity in descending pathways. Non-invasive neuromodulation approaches such as transcranial direct current stimulation (tDCS) and transcranial magnetic stimulation (TMS)—which regulate cortical excitability—have been widely adopted as adjunctive therapies for upper-limb functional rehabilitation. Through shared mechanisms including the modulation of neuroinflammation, glial activity, BDNF release, and the promotion of neural plasticity, they offer potential non-invasive strategies for neuroprotection and functional recovery after SCI(Markowska and Tarnacka, 2024). Further animal studies have revealed that tDCS can promote motor recovery in SCI mice by restoring neuronal synchrony in the brain and suppressing excessive microglial activation in the spinal cord (Oishi et al., 2024). Additionally, high-frequency repetitive TMS (HF-rTMS) has been shown to enhance functional recovery by inhibiting Cx43 expression, modulating autophagic flux, and activating the mTOR signaling pathway (Zhang et al., 2024b). Meanwhile, transcranial alternating current stimulation (tACS) demonstrates broad potential in neuroprotection, structural repair, and functional recovery via multiple mechanisms, such as regulating the cGMP-PKG pathway and inhibiting ferroptosis (Huang et al., 2025).

Clinical studies have provided mechanistic insights into how neuromodulation techniques can enhance SCI rehabilitation. Research shows that applying tDCS over the motor cortex during robot-assisted gait training specifically enhances activity in key brain regions, such as the supplementary motor area (SMA) and primary motor cortex (M1), and that this increase in neural activity correlates positively with actual improvements in patients’ walking ability (Coelho et al., 2025). This principle is illustrated in clinical cases: patients with incomplete SCI who received early, targeted rTMS treatment showed significant improvements in motor function, pain levels, and activities of daily living (Yang N. et al., 2025). However, maintaining the therapeutic effects of neuromodulation remains challenging. Studies indicate that even combined transcranial magnetic stimulation and transspinal direct current stimulation only temporarily enhance corticospinal excitability, with effects often diminishing after cessation of stimulation (Lin et al., 2023). This suggests that short-term neuromodulation alone may be insufficient to induce stable neural reorganization. Crucially, emerging advanced technologies (such as EES and BSIs) are not designed to replace conventional rehabilitation, but rather to act as its neurophysiological catalyst. While standard physical therapy often fails in severe SCI due to insufficient volitional descending drive, neuromodulation temporarily elevates the excitability of spinal circuits, enabling them to process residual signals. Therefore, this physical modality must be strictly articulated with active, task-specific rehabilitation training; only when bio-electronic stimulation and volitional training are closed-looped can regenerating axons successfully consolidate into stable, functional neural networks (circuit rewiring). Although long-term efficacy requires optimized protocols, current evidence supports an integrated approach combining neuromodulation with rehabilitation training. A meta-analysis further indicates that non-invasive electromagnetic neuromodulation combined with rehabilitation effectively improves upper limb motor function in patients with cervical SCI, highlighting the clinical value of this combined intervention model (Hernandez-Navarro et al., 2025).

In addition to non-invasive neuromodulation techniques, more targeted invasive strategies offer new directions for SCI rehabilitation. Preliminary clinical studies in animal models and two human SCI patients have shown that deep brain stimulation (DBS) targeting the lateral hypothalamic area (LHA) can improve walking ability. When combined with rehabilitation training, DBS may further promote neural circuit reorganization, leading to sustained functional recovery (Cho et al., 2024). Ji et al. (Ji et al., 2025) found that the LHA relays through the pontine reticular formation oral part (PnO), forming an indirect LHA–pons–spinal cord pathway that regulates the initiation of motivated movement. Building on this mechanism, they developed a “gated” DBS approach triggered in real time by motor-cortical intention signals. This technique can precisely activate the pathway and has been shown to significantly promote the recovery of hindlimb motor function in paralyzed mice after SCI.

Brain–computer interface (BCI) is reshaping motor restoration after spinal cord injury by extending its role from assistive replacement toward intention-driven neuromodulation and activity-dependent neural remodeling. Early research primarily focused on brain–machine interfaces that decoded motor cortical signals to control external robotic devices. The most transformative advancement to date is the closed-loop brain–spine interface (BSI), which uses a fully implanted “digital neural bypass” to translate recorded movement intentions into real-time electrical stimulation of specific spinal segments, thereby functionally reconnecting supraspinal commands with sublesional motor circuits (Lorach et al., 2023; Zou et al., 2023). A parallel strategy is brain-controlled functional electrical stimulation, where decoded intentions directly govern multichannel electrical stimulation to activate paralyzed muscles (Chen et al., 2023). The success of these technologies relies on several key breakthroughs including stable high-signal-to-noise neural recordings, bidirectional closed-loop modulation based on movement feedback, rapid-calibration algorithms, and their integration with intensive, task-specific rehabilitation training.

At the clinical level, early studies have shown that BSI technology can restore volitionally controlled walking in selected patients with chronic severe SCI, although current evidence remains limited to highly specialized settings and small patient cohorts (Lorach et al., 2023). Concurrently, brain-controlled electrical stimulation for upper limb function has successfully restored activities of daily living, such as grasping, in certain patients (Mokienko, 2024; Zhang H. et al., 2024). Recent advances include a “three-in-one” minimally invasive brain-spinal interface technology proposed by a team at Fudan University in 2025, which allows for single-session surgical implantation and enabled patients to resume leg movement within 24 h. Furthermore, a separate 5-year follow-up study confirmed the long-term safety and stability of a fully implantable BCI system, supporting its suitability for home use (Davis et al., 2025). In a related development, Neuralink has implanted devices in at least seven patients as of 2025, allowing them to operate electronic devices through thought, with plans to further expand into areas such as speech restoration.

In addition to restoring motor function, bidirectional BCIs offer a novel approach to sensory recovery. By delivering tactile signals back to the brain’s sensory cortex, these systems allow patients to re-perceive the touch of external objects, thereby improving the precision of their movements. To further enhance rehabilitation outcomes, BCIs are being deeply integrated with other advanced technologies: virtual reality (VR) provides immersive training environments, artificial intelligence (AI) improves the accuracy and adaptability of neural decoding, and flexible electrodes enhance biocompatibility and long-term stability. Furthermore, integrating synchronized visuo-auditory VR with wearable tactile and thermal sleeve feedback shifts the paradigm from simple visual immersion to comprehensive multimodal stimulation. This integrated approach actively remodels altered body representations by generating consistent, high-comfort embodiment experiences across body properties, volitional control, and tactile domains, which successfully mitigates chronic neuropathic pain and refines gait length perception during motor imagery training (Pais-Vieira et al., 2022). Hybrid rehabilitation systems that combine BCIs, electrical stimulation, and exoskeletons are paving the way for personalized closed-loop rehabilitation as an emerging clinical strategy.

In summary, BCI technology is advancing along three key trajectories: refining the precision of motor decoding, enhancing the biocompatibility of implantable materials, and accelerating the translation of laboratory findings into standardized clinical treatments. It is increasingly emerging as a pivotal rehabilitative tool for promoting neural remodeling and restoring sensory and motor functions (Table 3).

**TABLE 3:** Classification and clinical efficacy of neuromodulation techniques.

Neuromodulation technique	Stimulation target/Method	Invasiveness	Primary functions restored	Efficacy characteristics & durability	Target population
EES	Lumbar/Sacral dorsal roots	Yes (surgically implanted)	Lower limb standing/walking, bladder/bowel control, sexual function	Significant effect, requires continuous stimulation to maintain	Chronic complete/incomplete SCI
tcSCS	Percutaneous surface stimulation	No	Lower limb motor function, muscle tone regulation	High safety, effect weaker than EES, often combined with rehab	Incomplete SCI during rehabilitation
DBS	Lateral hypothalamic area (LHA)	Yes (intracranial implant)	Movement initiation, walking ability	Experimental, combined rehab may promote sustained recovery	Specific chronic SCI (exploratory)
tDCS	Motor cortex	No	Upper limb function, gait coordination	Short-term enhancement of cortical excitability, requires repeated sessions	Subacute/Chronic SCI as rehab adjunct
TMS	Motor cortex/Spinal cord	No	Upper limb motor function, neuropathic pain	Modulates corticospinal connectivity, relatively durable effect	Chronic SCI with neurological dysfunction
BSI	Motor cortex → spinal stimulation	Yes (fully implanted)	Volitional control of lower limb walking, hand grasping	Restores natural movement patterns; promotes sustained neurological improvement and neural remodeling	Severe chronic SCI (clinical trials)

## 8 Conclusions and perspectives

Over the past few decades, significant progress has been made in research focused on the repair and regeneration of SCI. However, achieving complete functional recovery through monotherapy remains a formidable challenge, primarily due to the intrinsic difficulty of regeneration within the central nervous system and the complex pathological microenvironment post-injury. To overcome these hurdles, the research focus has gradually shifted from solely promoting axonal regeneration toward a more comprehensive strategy involving neural circuit reconstruction and microenvironmental remodeling.

Current therapeutic strategies for spinal cord injury repair include stem cell therapy, extracellular vesicles, various biomaterials, and neuromodulation techniques, each exhibiting distinct characteristics in terms of mechanisms, advantages, limitations, and clinical translation stages. Based on this comparison, the major future research directions and translational challenges are summarized below (Table 4). Regarding stem cell therapy, we discuss the paradigm shift from traditional cell replacement towards microenvironmental modulation based on “paracrine” mechanisms, as well as the preliminary clinical applications of iPSCs and NPCs. Concerning EVs, this “cell-free” therapeutic approach demonstrates considerable potential in modulating neuroinflammation and promoting angiogenesis, attributed to its low immunogenicity and ability to cross the blood-spinal cord barrier. In the domain of biomaterials, we review how natural and synthetic biomaterials (e.g., hydrogels, conductive scaffolds, nanomaterials) serve as physical scaffolds and drug delivery vehicles to provide a more favorable microenvironment for neural regeneration. Furthermore, the rapid advancement of neuromodulation technologies, such as epidural electrical stimulation and brain-computer interfaces, offers novel clinical tools for activating residual neural circuits and restoring motor function.

**TABLE 4:** Comparison of major strategies for spinal cord injury repair.

Therapeutic strategy	Representative agents/Cells/Technologies	Main advantages	Current limitations/Challenges	Clinical translation stage
Stem cell therapy	MSCs, NSCs, iPSCs, SCs	Multi-mechanistic synergy (immunomodulation, neurotrophy, angiogenesis); safety established for some types	Low cell survival rate; tumorigenic risk; difficulties in standardized production; controversy over transplantation timing	Early clinical translation (multiple phase I/II studies across selected cell types)
Extracellular vesicles (EVs)	MSC-EVs, NSC-EVs; engineered EVs	Low immunogenicity; ability to cross blood-spinal cord barrier; cell-free therapy minimizes risks	Lack of standardized preparation; unclear *in vivo* pharmacokinetics; suboptimal targeting efficiency	Early preclinical/Exploratory clinical
Inorganic nanomaterials	AuNPs, CeONPs, SeNPs, ZnO NPs	Good biocompatibility; stability; multifunctionality	Unknown long-term toxicity; lack of clinical data	Preclinical research (animal studies)
Organic polymeric/Lipid nanomaterials	Polymeric micelles, LNPs, dendrimers	Biodegradable; high degree of functionalization; can encapsulate various therapeutics	Challenges in standardized preparation; unclear long-term safety	Primarily preclinical, few early clinical
Natural biomaterials	Collagen, GelMA, hyaluronic acid, decellularized scaffolds	Excellent biocompatibility Low immunogenicity; contain cell recognition sites	Poor mechanical properties; difficult to control degradation rate	Some materials clinically used in other medical contexts; complex scaffolds in preclinical
Synthetic biomaterials/Peptides	Self-assembling peptides, PCL, conductive hydrogels	Precisely controllable properties; strong mechanical performance; scalable production	Lack of intrinsic bioactivity; degradation products may cause inflammation	Preclinical research
Invasive neuromodulation	EES, DBS, BSI	Precise modulation; functional recovery clinically demonstrated	Surgical risks; high cost of devices; requires individualized parameter tuning	Clinical trials/Specialized clinical application in selected centers
Non-invasive neuromodulation	tDCS, TMS, transcutaneous spinal cord stimulation	Non-invasive; easily combined with rehabilitation; low risk	Limited stimulation depth; short-lived effects	Adjunct therapy/Rehabilitation application

Although the aforementioned strategies have shown promising results in basic research and early-stage clinical trials, several critical issues must be addressed, and future directions explored, to enable widespread clinical translation:

First, deepening multimodal synergistic therapeutic strategies. Analogous to the insufficiency of a single growth factor in complex tissue repair, monotherapies for SCI often yield limited efficacy. Future research should place greater emphasis on “combination therapies.” For instance, integrating stem cells or exosomes into functionalized biomaterials (e.g., conductive hydrogels or immunomodulatory scaffolds), combined with neuromodulation techniques (e.g., electrical stimulation), to construct an integrated “biological-engineering-physical” repair system. Such synergistic approaches can not only provide physical support and biochemical cues for cell growth but also mimic the physiological environment through electrical signals, thereby maximizing the efficiency of neural regeneration.

Second, developing smart responsive and precision delivery systems. Current therapeutic delivery methods still lack spatiotemporal precision in controlling the injury microenvironment. Inspired by intelligent hydrogel delivery systems, future SCI repair materials should be engineered with responsiveness to pathological cues (e.g., elevated reactive oxygen species, enzyme overexpression, pH changes), enabling on-demand release of therapeutic agents or factors. Furthermore, to address the staged nature of the SCI pathological process (anti-inflammatory in the acute phase and pro-regenerative in the chronic phase), the development of multi-stage delivery systems capable of sequentially releasing distinct therapeutic molecules will be crucial for enhancing treatment efficacy.

Third, advancing clinical translation standards and safety assessment. Many cutting-edge therapies, particularly those involving stem cells and nanomaterials, still face challenges in scalable production and quality control. Establishing standardized cell manufacturing protocols, defining the active component criteria for EVs, and unifying production standards for biomaterials are imperative. Concurrently, rigorous long-term safety assessments of novel therapeutic strategies are essential, particularly concerning implant biocompatibility, the tumorigenic risk of stem cells, and immune responses, to ensure a smooth transition from the laboratory to the clinic.

Fourth, exploring personalized and precision rehabilitation. Spinal cord injury is highly heterogeneous. With advances in 3D bioprinting technology and patient-specific iPSC techniques, future developments may enable the construction of personalized transplantation scaffolds tailored to the patient’s injury morphology and biological characteristics. Simultaneously, combining neuroimaging and biomarker monitoring to tailor individualized neuromodulation parameters and rehabilitation training regimens will be a vital pathway towards achieving optimal functional recovery.

In summary, the repair of spinal cord injury constitutes a complex systems engineering challenge spanning biology, materials science, medicine, and engineering. Through interdisciplinary integration, continued optimization of combinatorial therapeutic strategies, and the establishment of rigorous clinical translation standards, progress may be made in addressing key barriers to central nervous system regeneration and improving outcomes for patients with spinal cord injury.

## Glossary

SCI: Spinal Cord Injury
CNS: Central Nervous System
ASIA: American Spinal Injury Association
NSC: Neural Stem Cell
NPC: Neural Progenitor Cell
iPSC: Induced Pluripotent Stem Cell
MSC: Mesenchymal Stem Cell
OPC: Oligodendrocyte Precursor Cell
SC: Schwann Cell
hiPSC-NS/PC: human iPSC-derived Neural Stem/Progenitor Cell
BDNF: Brain-Derived Neurotrophic Factor
GDNF: Glial Cell Line-Derived Neurotrophic Factor
VEGF: Vascular Endothelial Growth Factor
HGF: Hepatocyte Growth Factor
EV: Extracellular Vesicle
GPX4: Glutathione Peroxidase four
CCL2: C-C Motif Chemokine Ligand two
GelMA: Gelatin Methacryloyl
AuNP: Gold Nanoparticle
SeNP: Selenium Nanoparticle
CeONP: Cerium Oxide Nanoparticle
MNP: Magnetic Nanoparticle
SOD: Superoxide Dismutase
HA: Hyaluronic Acid
SLN: Solid Lipid Nanoparticle
LNP: Lipid Nanoparticle
PCL: Polycaprolactone
EGFR: Epidermal Growth Factor Receptor
ASCM: Acellular Spinal Cord Matrix
PnO: Pontine Reticular Formation Oral part
SCS: Spinal Cord Stimulation
EES: Epidural Electrical Stimulation
tcSCS: Transcutaneous Spinal Cord Stimulation
tDCS: Transcranial Direct Current Stimulation
TMS: Transcranial Magnetic Stimulation
rTMS: Repetitive Transcranial Magnetic Stimulation
tACS: Transcranial Alternating Current Stimulation
BCI: Brain-Computer Interface
BSI: Brain-Spinal Interface
DBS: Deep Brain Stimulation
LHA: Lateral Hypothalamic Area
VR: Virtual Reality
AI: Artificial Intelligence
RCD: Regulated Cell Death
MQC: Mitochondrial Quality Control
DAM: Disease-Associated Microglia
LRAs: Lesion-Remote Astrocytes
epNSPCs: Ependymal Neural Stem/Progenitor Cells
enTsOrgs: Engineered Thoracic Spinal Cord Organoids
TNF-α: Tumor Necrosis Factor-alpha
IL-1β: Interleukin-1 beta
IL-10: Interleukin-10
TGF-β: Transforming Growth Factor-beta
PGE2: Prostaglandin E2
AMPAR: α-amino-3-hydroxy-5-methyl-4-isoxazolepropionic acid receptor
Htt: Huntingtin
TrkC: Tropomyosin receptor kinase C
PTCH1: Patched one
SHH: Sonic hedgehog
HDAC6: Histone deacetylase six
RIPK1: Receptor-interacting serine/threonine-protein kinase one
RIPK3: Receptor-interacting serine/threonine-protein kinase three
PTEN: Phosphatase and tensin homolog
AKT: Protein kinase B
mTOR: Mechanistic target of rapamycin
VGF: VGF nerve growth factor inducible
PTP-σ: Protein tyrosine phosphatase-sigma
ROCK: Rho-associated protein kinase
PI3K: Phosphoinositide 3-kinase
Cx43: Connexin 43
cGMP-PKG: Cyclic guanosine monophosphate-protein kinase G
SDC4: Syndecan four
LRIG3: Leucine-rich repeats and immunoglobulin-like domains protein three
dECM: Decellularized Extracellular Matrix
ECM: Extracellular Matrix
HAMA: Hyaluronic Acid Methacryloyl
CNT: Carbon Nanotube
PEG: Polyethylene Glycol
PLGA: Poly(lactic-co-glycolic acid)
BP: Black Phosphorus
ZnO NP: Zinc Oxide Nanoparticle
CBD-NT3: Collagen-binding domain-neurotrophin-3
TAT: Trans-activator of transcription
RVG: Rabies virus glycoprotein
RGD: Arginylglycylaspartic acid
CSPGs: Chondroitin Sulfate Proteoglycans
HUCMSC: Human Umbilical Cord Mesenchymal Stem Cell
CPGs: Central Pattern Generators
SMA: Supplementary Motor Area
M1: Primary Motor Cortex
PMOA: Primary Mode of Action
OCP: Office of Combination Products
CBER: Center for Biologics Evaluation and Research
CDRH: Center for Devices and Radiological Health
BLA: Biologics License Application
NMPA: National Medical Products Administration
cGMP: current Good Manufacturing Practices
CMC: Chemistry, Manufacturing, and Controls
ClisQAs: Critical Quality Attributes
